# The value of blood lactate kinetics in critically ill patients: a systematic review

**DOI:** 10.1186/s13054-016-1403-5

**Published:** 2016-08-13

**Authors:** Jean-Louis Vincent, Amanda Quintairos e Silva, Lúcio Couto, Fabio S. Taccone

**Affiliations:** Department of Intensive Care, Erasme Hospital, Université Libre de Bruxelles, Route de Lennik 808, 1070 Brussels, Belgium

## Abstract

**Background:**

The time course of blood lactate levels could be helpful to assess a patient’s response to therapy. Although the focus of published studies has been largely on septic patients, many other studies have reported serial blood lactate levels in different groups of acutely ill patients.

**Methods:**

We performed a systematic search of PubMed, Science Direct, and Embase until the end of February 2016 plus reference lists of relevant publications. We selected all observational and interventional studies that evaluated the capacity of serial blood lactate concentrations to predict outcome. There was no restriction based on language. We excluded studies in pediatric populations, experimental studies, and studies that did not report changes in lactate values or all-cause mortality rates. We separated studies according to the type of patients included. We collected data on the number of patients, timing of lactate measurements, minimum lactate level needed for inclusion if present, and suggested time interval for predictive use.

**Results:**

A total of 96 studies met our criteria: 14 in general ICU populations, five in general surgical ICU populations, five in patients post cardiac surgery, 14 in trauma patients, 39 in patients with sepsis, four in patients with cardiogenic shock, eight in patients after cardiac arrest, three in patients with respiratory failure, and four in other conditions. A decrease in lactate levels over time was consistently associated with lower mortality rates in all subgroups of patients. Most studies reported changes over 6, 12 or 24 hrs, fewer used shorter time intervals. Lactate kinetics did not appear very different in patients with sepsis and other types of patients. A few studies suggested that therapy could be guided by these measurements.

**Conclusions:**

The observation of a better outcome associated with decreasing blood lactate concentrations was consistent throughout the clinical studies, and was not limited to septic patients. In all groups, the changes are relatively slow, so that lactate measurements every 1–2 hrs are probably sufficient in most acute conditions. The value of lactate kinetics appears to be valid regardless of the initial value.

## Background

Since the early studies by Weil and others [1–3], blood lactate concentrations have been used widely as a marker of altered tissue perfusion in critically ill patients [4]. In physiological conditions, about 1500 mmol of lactate is produced daily from various organs, including the muscle, the intestine, the red blood cells, the brain, and the skin [5]. Lactate is metabolized by the liver (about 60 %), the kidneys (about 30 %), and other organs [5]. The normal blood lactate concentration is around 1 mEq/l [6]. Even minor increases in lactate concentrations to >1.5 mEq/l are associated with higher mortality rates [6, 7]. The exact pathophysiologic mechanisms of hyperlactatemia have been much debated, because the condition does not always simply reflect the development of anaerobic metabolism [8]. In sepsis in particular, metabolic alterations can contribute to elevated blood lactate concentrations, including increased glycolysis, catecholamine-stimulated Na–K pump activity, alterations in pyruvate dehydrogenase activity, and reduced lactate clearance primarily as a result of liver hypoperfusion. Regardless of these mechanisms, hyperlactatemia is a hallmark characteristic of shock states [4, 9] and the degree of increase in lactate concentrations is directly related to the severity of the shock state and to mortality rates [10, 11].

**Fig. 1:**
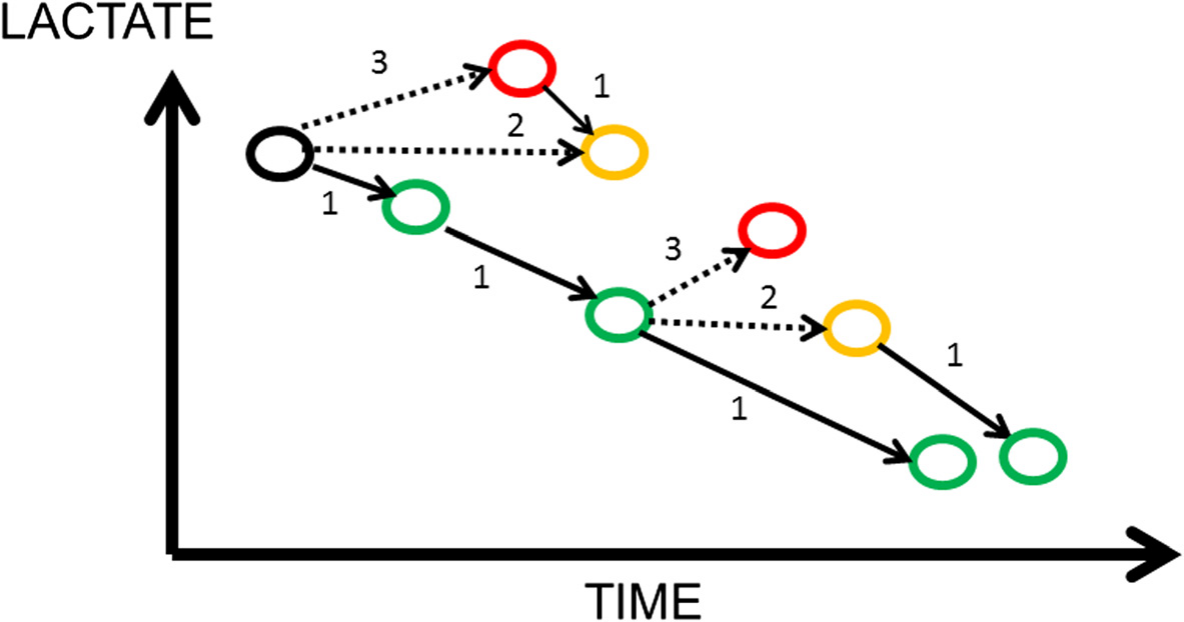
Schematic showing some of the possible evolutions of blood lactate levels over time: decreasing (*1*), remaining stable (*2*), or increasing (*3*). *Dashed lines* represent an unfavorable course and suggest the need for treatment to be reviewed, if this has not already been done, because the current management is likely ineffective

As for the blood levels of any substance, elevated lactate levels can be the result of increased production, reduced elimination, or both. A dynamic evaluation of serial lactate concentrations may thus be more informative than a single value. This concept of repeating blood lactate concentrations over time as an indicator of response to therapy was first proposed in 1983 [12], based on an idea raised after a publication by Orringer et al. in 1977 [13] showing that the decrease in lactate levels after cessation of grand mal seizures was actually quite rapid, with a half-life of about 50 % in 1 hr. Many studies have since emphasized that changes in lactate over the first hrs of treatment may represent a valuable monitoring tool. Some studies have even proposed integrating changes in lactate concentrations as a target in therapeutic protocols [14–17] or including them as one of the sepsis resuscitation “bundles” [18]. A number of investigators have used the term “lactate clearance” to describe decreasing lactate levels, but this is incorrect for two reasons. The first is that the changes in lactate concentrations over time reflect changes in production and in elimination. The decrease in lactate over time may reflect decreased (over)production more than increased clearance by the liver and other organs [19, 20]. The specific study of lactate clearance would require intravenous injection of radiolabeled lactate, as has been done in several studies [21, 22]. The second reason why use of the term is incorrect is that “clearance” or “elimination” implies a progressive normalization of blood lactate concentrations, which is too simplistic. Blood lactate concentrations can have a complex evolution and may even increase over time (Fig. 1), a situation that one should then call “negative lactate clearance”.

We performed a literature search on this subject to address several questions. First, is the observation of a better prognosis with decreasing lactate concentrations a consistent finding in all types of critically ill patient? Second, although some studies have suggested that repeated lactate measurements may be particularly useful in sepsis, can similar observations be made in other acute disease states or even in heterogeneous groups of critically ill patients? Third, how fast should lactate concentrations decrease in optimal conditions and is there any particular time interval that could be recommended? Fourth, some studies in emergency medicine considered only patients with lactate values > 4 mEq/l as an at-risk population, but is this approach valid? In other words, is the study of lactate kinetics more useful when lactate concentrations exceed a given value?

## Methods

We searched databases of PubMed, Science Direct, and Embase until the end of February 2016 to identify studies that evaluated the capacity of serial blood lactate concentrations to predict outcome, using the search terms “Lactate levels” OR “lactate clearance” AND “shock” OR “critically ill” AND “mortality”. We included original prospective or retrospective clinical studies. There was no restriction based on language. We excluded studies in pediatric populations, experimental studies, case reports, and studies that did not report changes over time in lactate values or relationship of changes in lactate concentrations to all-cause mortality rates. We had no restriction on the initial location in the hospital (e.g., ICU, trauma unit, emergency room, operating room). We also checked the reference lists of included articles to capture any references missed during the search. We classified the different adult populations into general ICU patients, general surgical ICU patients, cardiac surgery patients, trauma patients, patients with sepsis, patients with cardiogenic shock, post-cardiac arrest patients, patients with respiratory failure, and others.

## Results

**Fig. 2:**
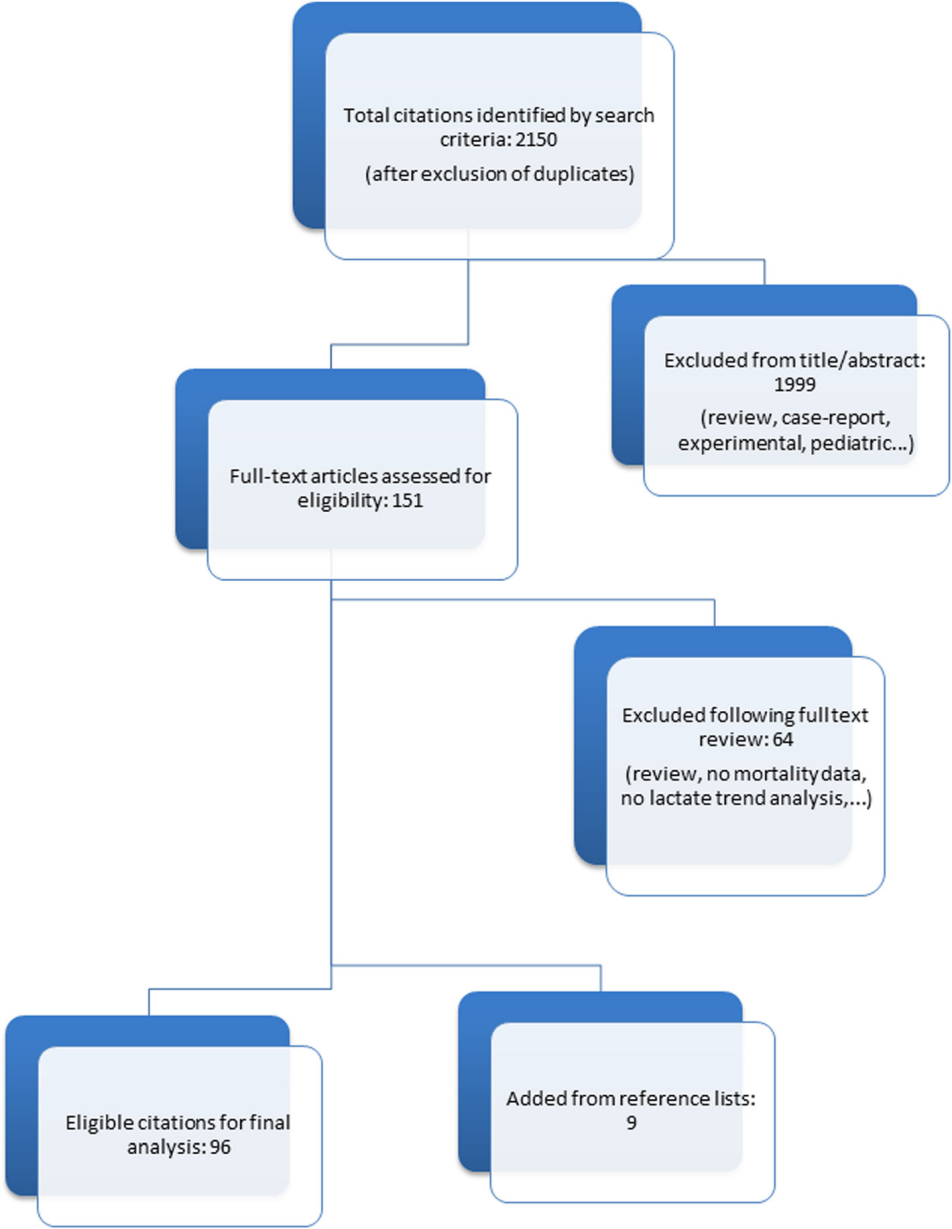
Prisma diagram

A total of 96 studies met our inclusion criteria (Fig. 2, Table 1).

**Table 1:** Included studies according to population type

	First author, year [reference]	Number of patients	Study design	Initial minimum lactate for patient inclusion	Timing of measurements	Suggested time interval	Comments
	General ICU/emergency department						
	*Observational*						
1.	Vincent, 1983 [12]	17	Prospective	≥4	Every 20 min during first 2 h of ICU treatment	1 h	Decrease >10 % associated with survival
2.	Cowan, 1984 [23]	30	Prospective	–	3 h, 24 h	3 h	Change in lactate predictive of outcome but less so than simple hemodynamic variables
3.	Suistomaa, 2000 [24]	100	Prospective	–	Every 2 h for 24 h	6 h	Failure to decrease lactate at 6 h associated with higher mortality
4.	Jansen, 2008 [31]	106	Prospective	–	Variable (at ambulance pickup and at ER arrival)	—	Decrease in lactate independently associated with decreased hazard of death
5.	Wang, 2009 [25]	101	NR	≥2	12 h, 24 h	12 h	Decrease ≤10 % associated with increased mortality
6.	Jansen, 2009 [26]	394	Prospective	–	12 h, 24 h	12 h	Decrease in lactate only of prognostic value in patients with sepsis
7.	Krishna, 2009 [27]	50	Prospective	–	12 h, 24 h, 36 h	24 h, 36 h	Decreasing levels associated with survival
8.	Soliman, 2010 [28]	433	Prospective	–	24 h, 48 h	24 h	Higher lactate concentrations at 24 and 48 h after admission associated with decreased survival
9.	Nichol, 2010 [6]	7155	Retrospective	–	Variable	24 h	Time-weighted average lactate over 24 h independent predictor of mortality
10.	Nichol, 2011 [10]	5041	Retrospective	–	Variable	24 h	Time-weighted average lactate and change in lactate over 24 h independent predictors of hospital mortality
11.	van Beest, 2013 [29]	2251	Retrospective	–	Variable	6 h	Normalization of lactate <6 h after ICU admission associated with better hospital survival than normalization of lactate >6 hrs
12.	Zhang, 2014 [30]	6291	Retrospective	>2	Variable	Variable	Normalization and speed of normalization related to outcome
13.	Haas, 2016 [11]	400	Retrospective	>10	Variable	12 h	No decrease in lactate over 12 h associated with increased mortality
	*Interventional*						
14.	Jansen, 2010 [15]	348	Prospective	≥3.0	2 h	8 h	Objective was to decrease lactate by 20 % or more per 2 h for the initial 8 h of ICU stay. Lactate-guided therapy was independently associated with reduced hospital mortality
	Surgical ICU						
15.	McNelis, 2001 [32]	95	Retrospective	–	8-h intervals until lactate normalized	Variable	Time to lactate normalization predictive of outcome
16.	Husain, 2003 [33]	137	Retrospective	–	Variable	Variable	Time to lactate normalization independent predictor of mortality
17.	Meregalli, 2004 [34]	44	Prospective	–	12 h, 24 h, 48 h	48 h	Blood lactate concentrations decreased with time in survivors, but remained stable in nonsurvivors
18.	Cardinal Fernandez, 2009 [35]	108	Prospective	>2	6 h	6 h	Decrease in lactate by >40 % associated with increased survival
19.	Ibrahim, 2013 [36]	322	Prospective	–	8 h, 16 h, 24 h	16 h	Percent change in blood lactate at 16 h independent predictor of postoperative mortality
	Cardiac surgery						
20.	Lindsay, 2013 [37]	1291	Retrospective	–	Variable	Variable	Longer predicted time to reach normal lactate (<1.5 mmol/l) associated with increased mortality
21.	Hajjar, 2013 [38]	502	Prospective	–	6 h, 12 h	6 h, 12 h	Failure to decrease lactate associated with major complications, including death
22.	Park, 2014 [39]	115	Retrospective	–	6 h, 12 h, 24 h	6 h, 12 h, 24 h	Lack of decrease in lactate predictive of mortality
23.	Lopez-Delgado, 2015 [40]	2935	Prospective	–	6 h, 12 h, 24 h	24 h	Later peak in lactate associated with higher hospital and long-term mortality
24.	Li, 2015 [41]	123	Retrospective	–	6 h, 12 h	12 h	Lactate decrease predictive of in-hospital mortality in patients receiving ECMO
	Trauma						
	*Observational*						
25.	Abramson, 1993 [42]	76	Prospective	–	8 h, 16 h, 24 h, 36 h, 48 h	24 h	Normalization of lactate by 24 h associated with 100 % survival
26.	Manikis, 1995 [43]	129	Retrospective	–	At least three times a day	Variable	Duration of hyperlactatemia correlated with the development of organ failure but not with mortality
27.	Holm, 2000 [44]	21	Prospective	–	12 h, 24 h, 48 h, 72 h	Variable	Decreasing lactate levels associated with survival
28.	Cerovic, 2003 [45]	98	Prospective	–	Twice daily during first 2 days and once daily during next 3 days	Variable	Reduced lactate levels in survivors
29.	Kamolz, 2005 [46]	166	Prospective	–	Variable	24 h	Higher mortality in patients with initial lactate > 2 mmol/l if lactate not normalized at 24 h
30.	Billeter, 2009 [47]	1032	Retrospective	–	Variable	24 h	Delayed or absent decrease in lactate associated with infectious complications but not mortality
31.	Regnier, 2012 [48]	281	Prospective	–	2 h, 4 h	2 h	Early normalization of lactate independent predictor of survival
32.	Dubendorfer, 2013 [49]	724	Retrospective	–	Variable	Variable	In patients without traumatic brain injury, decrease in lactate impaired in nonsurvivors
33.	Odom, 2013 [50]	623	Retrospective	≥4	6 h	6 h	Lower decrease in lactate at 6 h independently predictive of increased risk of death
34.	Heinonen, 2014 [51]	610	Retrospective	–	Variable	Variable	Failure to normalize lactate associated with increased mortality
35.	Freitas, 2015 [52]	117	Retrospective	–	6 h	6 h	No correlation between decrease in lactate and mortality
36.	Dezman, 2015 [53]	3887	Retrospective	≥3	Variable	Variable	No decrease in lactate independent predictor of 24-h mortality
	*Interventional*						
37.	Blow, 1999 [55]	79	Retrospective	–	Variable	24 h	Failure to decrease lactate associated with increased mortality
38.	Claridge, 2000 [56]	364	Prospective	–	Variable	12 h	Increase in infections, length of stay, and mortality if lactate did not normalize by 12 h
	Sepsis						
	*Observational*						
39.	Bakker, 1991 [57]	48	Prospective	>2	Variable	Variable	Only survivors had a significant decrease in blood lactate concentrations during the course of septic shock
40.	Friedman, 1995 [58]	35	Prospective	>2	4 h, 24 h	Variable	Lactate remained high in nonsurvivors and progressively decreased in survivors
41.	Bernardin, 1996 [59]	32	Prospective	–	24 h	24 h	Greater decrease in lactate in survivors
42.	Marecaux, 1996 [60]	38	Prospective	>2	24 h, 48 h	24 h, 48 h	Greater decrease in lactate in survivors
43.	Bakker, 1996 [61]	87	Prospective	>2	Variable	Variable	Duration of lactic acidosis best discriminant of survival
44.	Kobayashi, 2001 [62]	22	Prospective	–	Every 4 hours for 4 days	Variable	Decrease in lactate associated with survival
45.	Nguyen, 2004 [63]	111	Prospective	–	6 h	6 h	Decrease in lactate ≥10 % associated with lower 60-day mortality
46.	Nguyen, 2007 [64]	330	Prospective	–	Variable	6 h	Decreased odds ratio for mortality in patients with decreased lactate
47.	Phua, 2008 [65]	72	Prospective	–	24 h, 48 h	24 h	Increase in lactate predictive of mortality
48.	Yang, 2009 [66]	105	Prospective	–	6 h, 24 h, 72 h	6 h	Decrease in lactate at 6 h ≥30 % was independent predictor of survival
49.	Arnold, 2009 [67]	166	Retrospective	–	6 h	6 h	Lactate decrease by less than 10 % independent predictor of in-hospital death
50.	Nguyen, 2010 [68]	220	Retrospective	–	6 h	6 h	Larger decrease in lactate associated with decreased mortality up to 12 months
51.	Nguyen, 2011 [18]	556	Prospective	–	12 h	12 h	Any decrease in lactate within 12 h from baseline or an initial lactate <2 mmol/l independently associated with reduced mortality
52.	Puskarich, 2012 [69]	203	Retrospective analysis of data from [16]	–	2 h, 4 h, 6 h	6 h	≥10 % decrease in lactate during resuscitation associated with decreased mortality
53.	Zanaty, 2012 [70]	53	Prospective	–	6 h	6 h	<15 % decrease in lactate independent predictor of mortality
54.	Puskarich, 2013 [71]	187	Retrospective analysis of data from [16]	–	At least two lactate measurements in first 6 h	6 h	Lactate normalization in 6 h stronger independent predictor of survival than decrease in lactate by ≥50 %
55.	Walker, 2013 [72]	78	Retrospective	–	6 h	6 h	Decrease in lactate independently associated with mortality, with optimal cut-off of 36 %
56.	Liu, 2013 [73]	9190	Retrospective	≥2	4 h, 8 h, 12 h	12 h	Reduced mortality in patients with more than 60 % lactate improvement at 12 h.
57.	Marty, 2013 [74]	94	Prospective	–	6 h, 12 h, 24 h	24 h	Decrease in lactate at 24 h independently correlated to survival
58.	Park, 2014 [75]	25	Prospective	–	6 h, 12 h, 18 h, 24 h, 48 h	48 h	Normalization independent predictor of survival
59.	Permpikul, 2014 [76]	51	Prospective	–	6 h	6 h	Lactate decrease associated with reduced 28-day mortality
60.	Bao, 2015 [77]	94	Retrospective	–	3 h, 6 h, 24 h	24 h	24-h lactate decrease predictive of outcome
61.	Galbois, 2015 [78]	42	Prospective	–	6 h, 12 h, 18 h, 24 h	6 h	Lesser decrease in lactate associated with 14-day mortality
62.	Lee, 2015 [79]	109	Retrospective	>3.3	6 h, 24 h, 48 h	6 h, 24 h, 48 h	Decrease in lactate of <10 % in the first 6 h, 24 h, and 48 h independently associated with mortality
63.	Dettmer, 2015 [17]	243	Retrospective	≥4	Variable	Variable	Greater reduction in lactate associated with decreased 28-day mortality
64.	Lokhandwala, 2015 [80]	74	Retrospective	≥4	Variable	Variable	Lactate decrease < 4 mmol/l associated with increased hospital morality
65.	Wang, 2015 [81]	115	Prospective	–	6 h, 12 h, 18 h, 24 h	24 h	Lower lactate area score and percentage decrease in lactate associated with increased mortality
66.	Bhat, 2015 [82]	207	Retrospective	–	Variable	Variable	Higher mortality in patients with no decrease in lactate
67.	Chertoff, 2016 [83]	229	Retrospective	–	24-48 h	24-48 h	Lower decrease in plasma lactate 24–48 h after initiation of treatment was associated with higher 30-day mortality
68.	Drumheller, 2016 [84]	411	Retrospective	≥4	Variable	Variable	Decrease in lactate independently associated with decreased risk of death
69.	He, 2016 [85]	84	Prospective	–	8 h	8 h	Patients with lactate decrease ≥10 % had lower ICU mortality than those with lactate decrease <10 %
70.	Ha, 2016 [86]	208		–	6 h, 24 h	24 h	Low decrease in lactate at 6 and 24 h independently associated with hospital mortality, but 24-h lactate decrease had higher discriminatory power
71.	Bolvardi, 2016 [87]	90	Prospective	–	6 h	6 h	Lactate decrease <10 % associated with increased mortality
72.	Amir, 2016 [88]	202	Prospective	–	6 h	6 h	Lactate decrease ≥10 % not associated with mortality
	*Interventional*						
73.	Jones, 2010 [16]	300	Prospective	–	Variable	Variable	No differences in in-hospital mortality using management to normalize lactate compared with management to normalize ScvO_2_
74.	Tian, 2012 [89]	62	Prospective	–	Variable	48 h	28-day mortality rates lower in patients with 30 % decrease in lactate target than in those with 10 % decrease in lactate target and controls
75.	Yu, 2013 [90]	50	Prospective	–	3 h, 6 h, 72 h	6 h, 72 h	No differences in in-hospital mortality using management targeted at 10 % lactate decrease compared with management to normalize ScvO_2_
76.	Lyu, 2015 [91]	100	Prospective	–	1 h, 2 h, 3 h, 4 h, 5 h, 6 h	6 h	28-day mortality independently associated with lactate decrease <10 %
77.	Kuan, 2016 [92]	122	Prospective	≥3	Variable	3 h	Lactate decrease >20 % associated with decreased mortality
	Cardiogenic shock						
78.	Attana, 2012 [93]	51	Prospective	–	12 h	12 h	Decrease in lactate by <10 % predicts higher risk of death
79.	Attana, 2013 [94]	63	Prospective	–	12 h	12 h	Nonsurvivors had smaller decrease in lactate
80.	Park, 2014 [95]	96	Retrospective	–	Variable	48 h	Lactate decrease <70 % independent predictor of hospital mortality
81.	Guenther, 2014 [96]	41	retrospective	–	Variable	6 h	Increased lactate concentrations at 6 h associated with nonsurvival after ECMO
	Cardiac arrest						
82.	Kliegel, 2004 [97]	394	Retrospective	–	24 h, 48 h	48 h	Persistent hyperlactatemia predictive or poor prognosis
83.	Donnino, 2007 [98]	79	Retrospective	–	6 h, 12 h	12 h	Decrease in lactate independent predictor of hospital survival
84.	Arnalich, 2010 [99]	85	Prospective	–	6 h	6 h	Decrease in lactate significantly higher in 24-h survivors compared with nonsurvivors
85.	Le Guen, 2011 [100]	51	Prospective	–	1 h	1 h	Decrease in blood lactate >10 % significantly different in survivors and nonsurvivors treated with ECMO
86.	Starodub, 2013 [101]	199	Retrospective	–	6 h, 12 h, 24 h	12 h, 24 h	Change in lactate over time not predictive of survival but lower mean lactate levels at 12 and 24 h associated with increased survival
87.	Donnino, 2014 [102]	100	Prospective	–	12 h, 24 h	12 h	Greater percentage decrease independently associated with survival
88.	Riveiro, 2015 [103]	54	Prospective	–	6 h, 12 h, 24 h, 48 h, 72 h	6 h	Decrease in lactate predictive of 28-day survival
89.	Williams, 2016 [104]	167	Retrospective	–	Variable	4 h	More rapid decrease in lactate in survivors
	Respiratory failure						
90.	Zhao, 2010 [105]	110	Prospective	–	6 h	6 h	Lactate decrease ≥10 % associated with improved survival
91.	Wu, 2012 [106]	27	Prospective	–	12 h, 24 h, 48 h, 72 h	12 h, 24 h, 48 h, 72 h	Smaller decrease in lactate predictive of outcome
92.	Zang, 2014 [107]	43	Prospective	–	6 h	6 h	Decrease in lactate independent predictor of survival in patients treated by ECMO
	Others						
93.	Scott, 2010 [110]	95	Prospective	–	1 h, 2 h, 6 h, 24 h	2 h	Lactate decrease <15 % predictive of poor outcome (hospital mortality or endotracheal intubation) in patients with cardiorespiratory insufficiency
94.	Wu, 2011 [109]	222	Prospective	–	6 h	6 h	Lactate decrease of <24.8 % at 6 h associated with higher incidence of liver graft failure and mortality
95.	Lui, 2013 [108]	204	Prospective	≥2	12 h	12 h	Smaller decrease in lactate associated with increased mortality in patients with paraquat poisoning
96.	Mohamed, 2014 [111]	46	Prospective	–	8 h, 24 h	24 h	Mortality greater if <40 % decrease in lactate

*EMCO* extracorporeal membrane oxygenation, *ER* emergency room, *h* hours, *NR* not reported, *ScvO*_*2*_ central venous oxygen saturation

### General ICU patients

#### Observational studies

We identified 13 observational studies in heterogeneous critically ill populations [6, 10–12, 23–31]. All of these studies indicated that nonsurvivors had persistently higher lactate concentrations over time than survivors. Only one study [26] reported that lactate reduction during the first 24 hrs of ICU stay was useful only in septic patients, but not in patients with hemorrhage or other conditions.

The suggested optimal timing of lactate measurements was not precisely defined in several of the studies that evaluated the course of lactate concentrations over time. The studies that did include a time interval usually selected 6, 12 or even 24 hrs.

#### Interventional studies

An interventional trial of 348 patients by Jansen et al. [15] targeted a lactate decrease of at least 20 % in 2 hrs for the initial 8 hrs of treatment in ICU patients with an initial lactate ≥ 3 mEq/l. This strategy was associated with a lower mortality rate in the lactate-guided therapy group after adjustment for predefined risk factors (hazard ratio (HR), 0.61; confidence interval (CI), 0.43–0.87).

### Surgical patients

We identified five observational studies conducted in general surgical ICU patients [32–36]. Failure of lactate concentrations to decrease over time was associated with worse outcomes in all studies.

### After cardiac surgery

There were five observational studies in cardiac surgery patients [37–41], including two studies in patients treated with extracorporeal membrane oxygenation (ECMO) post cardiac surgery [39, 41]. All studies consistently demonstrated differences in changes in lactate concentration between survivors and nonsurvivors.

### Trauma patients

#### Observational studies

We identified twelve observational studies in trauma patients [42–53]. Three retrospective studies reported no association of change in lactate levels with mortality [43, 47, 52], although Manikis et al. [43] reported that the duration of hyperlactatemia was associated with the development of organ failure and Billeter et al. [47] noted that delayed or no reduction in blood lactate was associated with increased infectious complications. Several small studies used relatively long time intervals of 12–24 hrs [45, 54]. One study reported that repeated lactate after 2 hrs could be valuable [48] and a retrospective study proposed a time limit of 6 hrs [50].

#### Interventional studies

In a retrospective analysis of a small prospective cohort managed according to a protocol to normalize blood lactate levels, Blow et al. [55] reported that failure to normalize blood lactate levels (<2.5 mmol/l) was associated with increased morbidity and mortality. In an interventional study by Claridge et al. [56], patients were managed according to the same protocol targeted at reducing lactate levels to <2.4 mmol/l. Failure to achieve this target was associated with increased risk of infection, increased length of stay, and increased mortality.

### Patients with sepsis

#### Observational studies

We found thirty four observational studies in patients with sepsis [17, 18, 57–88]. One study reported that a decrease in lactate levels of ≥10 % was not associated with mortality [88], but this study was conducted in a low-resource setting, such that resuscitation may not have been optimal as acknowledged by the authors. Several studies reported that 6-hrly changes could be a useful guide [63, 64, 66, 67, 69–72, 78].

#### Interventional studies

One interventional study by Jones et al. [16] compared resuscitation based on lactate concentrations with a target of obtaining a >10 % decrease from the initial value with resuscitation based on achieving central venous oxygen saturation (ScvO_2_) ≥ 70 %; there were no differences in outcome between the two strategies. In an analysis of patients in this trial who had simultaneous lactate and ScvO_2_ measurements, Puskarich et al. [69] concluded that failure to achieve the target lactate decrease was associated with a worse prognosis than failure to achieve the ScvO_2_ target. In a small Chinese study [89], patients randomized to a 30 % decrease in lactate target had better 28-day survival than those randomized to a 10 % target or to control, and in another small study [90] there were no differences in in-hospital mortality using management targeted at a 10 % decrease in lactate compared with management to normalize ScvO_2_. Two other Chinese studies reported that patients randomized to lactate-directed therapy had improved outcomes [91, 92].

### Patients with cardiogenic shock

There were four studies in patients with cardiogenic shock [93–96], all showing that lactate concentrations decreased more in survivors than in nonsurvivors.

### After cardiac arrest

We identified eight observational studies [97–104] in post-cardiac arrest patients. All but one [101] of these studies demonstrated differences in changes in lactate concentration between survivors and nonsurvivors.

### Patients with acute respiratory failure

We found three observational studies in patients with acute respiratory failure [105–107], all showing that decreasing lactate levels were predictive of survival.

### Other conditions

Changes in lactate concentrations were also reported following paraquat poisoning [108], after liver transplantation [109], in patients with acute cardiorespiratory failure [110], and in patients with severe community-acquired pneumonia [111]. All studies indicated the value of repeated lactate concentrations in these patient populations.

## Discussion

Our literature review clearly supports the value of serial lactate measurements in the evaluation of critically ill patients and their response to therapy. This observation was similar across all studies and in all categories of patients, without being restricted to those with sepsis. We found only one study which suggested that evaluating the time course of lactate concentrations would be useful in sepsis patients but not in other conditions [26], and just five studies reporting no predictive effect of decrease in lactate levels over time on mortality [43, 47, 52, 88, 101] although two of these did suggest a relationship with morbidity outcomes [43, 47]. Repeated lactate concentrations can also help separate patients with complications, such as neurological complications after cardiac arrest [112, 113] or after surgery [38]. A meta-analysis of these data is complicated by the heterogeneity of the populations and the different timings of the measurements, but the data are very consistent across studies.

Increased lactate concentrations can be due to factors other than cellular hypoxia, so the decrease in blood lactate concentrations may not just be the result of improvements in cellular oxygen availability. For example, beta-adrenergic stimulation may contribute to increased lactate production [114]. A recent study indicated the reverse phenomenon; that is, the increase in lactate concentrations seen in patients with sepsis may be blunted in patients previously treated with beta-blocking agents [115]. The infusion of lactate-containing intravenous solutions may also potentially complicate the interpretation of blood lactate concentrations [116], although the amount of fluid infused must be very large to have such an effect [117]. A recent study also reported that lactate levels decrease more slowly in patients with a positive blood alcohol level, thus complicating evaluation of blood lactate levels in these patients [118].

Because lactate is primarily metabolized in the liver, liver dysfunction may alter lactate clearance. Thus, some studies have questioned whether blood lactate concentrations can be used to indicate tissue hypoperfusion in critically ill patients with hepatic dysfunction. However, patients with stable cirrhosis have normal lactate concentrations [8]. Kruse et al. [119] analyzed the incidence of hyperlactatemia in patients with liver disease and showed that lactic acidosis was associated with clinical evidence of shock and increased hospital mortality. Chiolero et al. [120] reported that major hepatectomy was not associated with any global changes in lactate clearance, although lactate half-life was prolonged. A recent experimental study indicated that liver hypoperfusion is unlikely to contribute to increased blood lactate concentrations [121]. In patients with paracetamol-induced acute liver failure, higher lactate concentrations were associated with more severe organ failure and mortality [122].

Some investigators have compared lactate and ScvO_2_ or combined the two measures. Lactate is usually a better prognostic marker [69]. But is it actually necessary to choose? In an interventional study in patients with sepsis, Jones et al. [16] reported no differences in outcomes for patients managed according to lactate concentrations or to ScvO_2_ values, but it is difficult to evaluate how these measurements really guided therapy because there were no differences in administered treatments during the first 72 hrs. In post-cardiac surgery patients, Polonen et al. [14] reported better outcomes when ScvO_2_ and lactate concentrations were targeted together than in control patients. The most convincing evidence in favor of lactate as a target comes from the study by Jansen et al. [15] in which outcomes were improved in patients treated to a target of a 20 % decrease in lactate concentrations. Nevertheless, the relatively slow changes in lactate make it difficult to interpret these results—the trend analysis is more a marker of effective treatment than a target in itself.

Although changes in blood lactate kinetics were clearly significant after 6 hrs in many studies and after 12 hrs in most, it is currently not possible to define the best time interval between lactate measurements. The normal reduction in lactate concentrations when overproduction of lactate abruptly ceases after grand mal seizures is about 50 % in 1 hr [13]. Although Levraut et al. [21] suggested that lactate clearance may be decreased in septic patients, Revelly et al. [22] reported similar values in patients with sepsis and in healthy volunteers.

The rate of lactate decrease in optimal treatment conditions is quite variable. In the best conditions, blood lactate concentrations decreased by more than 10 % in 1 hr in patients who responded rapidly to resuscitation [12] or by 10–20 % in 2 hrs [15]. A study by Hernandez et al. [123] suggested a >50 % decrease in lactate concentrations during the first 6 hrs of resuscitation in patients with septic shock. Although some systems now allow the quasi-continuous measurement of lactate concentrations, determinations every 1–2 hrs are probably sufficient; in the interventional study by Jansen et al. [15] the protocol was to measure blood lactate every 2 hrs. Even though serial blood lactate concentrations have been suggested to guide therapy, our review underlines that changes in lactate over time are relatively slow, taking place over hrs, and this may be too slow to guide therapy. Serial lactate concentrations should serve as a regular control, similar to how in the past a navigator would consult a compass from time to time to ensure that their boat was still heading in the right direction. If lactate concentrations do not normalize over time, the need for changes in therapy should be considered.

## Conclusion

Our systematic literature review has provided the following answers to our initial questions. First, observation of a better prognosis with decreasing lactate concentrations is consistent throughout the literature. Second, these observations are not specific to septic patients, but apply to all common situations of hyperlactatemia and in heterogeneous patient populations. Third, the changes are relatively slow, and it is difficult to provide recommendations about the speed of decrease in lactate concentrations in the best conditions. Clearly repeating measurements every 12 hrs can generally separate those who will do well from those who are likely to die, but shorter time intervals may be helpful. On the basis of our observations, we would recommend checking blood lactate concentrations as often as every 1–2 hrs in acute conditions. Fourth, the study of lactate kinetics appears to be valid regardless of the initial value and not only in patients with severe hyperlactatemia.

## Abbreviations

ECMO, extracorporeal membrane oxygenation; ScvO_2_, central venous oxygen saturation
